# Gut-Brain Axis: Understanding the Interlink Between Alterations in the Gut Microbiota and Autism Spectrum Disorder

**DOI:** 10.7759/cureus.88579

**Published:** 2025-07-23

**Authors:** Anushka P Mishra, Laura M Marrelli, Felicia T Bonner-Reid, Pallavi Shekhawat, Renée Toney, Ishmanjot K Benipal, Helga A Dias, Ayoub Kandi, Humza F Siddiqui

**Affiliations:** 1 Medicine, Era's Lucknow Medical College and Hospital, Lucknow, IND; 2 Psychiatry, Sapienza Università di Roma, Rome, ITA; 3 Medicine and Surgery, University of Medical Sciences of Manzanillo (Celia Sánchez Manduley), Manzanillo, CUB; 4 Obstetrics and Gynecology, Postgraduate Institute of Medical Sciences and Research (PGIMSR) and Employees' State Insurance (ESI) Model Hospital, Delhi, IND; 5 Medical Sciences, The University of the West Indies, Kingston, JAM; 6 Medicine, Dayanand Medical College and Hospital, Ludhiana, IND; 7 Medicine, Karwar Institute of Medical Sciences, Karwar, IND; 8 Medicine, Université d’Alger 1 - Benyoucef Benkhedda, Algiers, DZA; 9 Internal Medicine, Jinnah Postgraduate Medical Centre, Karachi, PAK

**Keywords:** autism spectrum disorder (asd), fecal microbiota transplant, gut-brain axis, gut dysbiosis, gut permeability

## Abstract

Autism spectrum disorder (ASD) is an umbrella term used for a complex neurobehavioral disorder. ASD is a multifactorial condition, with significant roles played by environmental, immunological, and genetic factors. The microbiota-gut-brain axis has been implicated in the pathophysiology of ASD in recent years.

This review article aims to explore the correlation between gut dysbiosis and autism, and its potential impact on management strategies. Gastrointestinal (GI) symptoms, including diarrhea, constipation, and bloating, are prevalent among children with ASD. These disorders are commonly linked to increased behavioral symptoms, such as social disengagement, anxiety, and irritability. Increased gut permeability, attributable to gut dysbiosis, plays a significant role in disrupting the gut-brain axis, which is coordinated by neurological, immunological, and endocrinological routes. Elevated levels of inflammatory cytokines, changes in the generation of neurotransmitters, and disturbances in gut-derived metabolites are all considered direct consequences of dysbiosis. Treatment options, including probiotics, prebiotics, fecal microbiota transplantation (FMT), and dietary changes, have shown promising results. However, the effectiveness and long-term safety of these therapies are still being studied. It is imperative to explore this perplexing interaction through further research to encourage clinicians to adopt therapeutic approaches targeting the gut microbiota in patients with ASD.

## Introduction and background

Autism spectrum disorder (ASD) is a heterogeneous group of neurodevelopmental diseases, serving as an umbrella term encompassing conditions such as autistic disorder, Asperger’s syndrome, pervasive developmental disorder, and childhood disintegrative disorder, among others. Manifestations of ASD generally begin in childhood, with the majority of cases being clinically diagnosed by the age of three [1]. The degree of affectation varies along the spectrum, ranging from mild to severe, and the characteristics differ significantly among individuals [2].

Generally, autistic children are categorized into three levels depending on severity: Level 1 (requiring support), Level 2 (requiring substantial support), and Level 3 (requiring very substantial support). The more typical presentations encompass core symptoms characterized by deficits in social reciprocity and communication, along with behavioral challenges, including unusual, restrictive, and repetitive stereotypical behavioral patterns and interests [1-3].

ASD is one of the most common neurodevelopmental disorders globally, with a significant increase in both incidence and prevalence. A study revealed a 175% increase in ASD diagnoses, from 2.3 per 1,000 in 2011 to 6.3 per 1,000 in 2022, among five-to-eight-year-olds [3]. In 2020, the Centers for Disease Control and Prevention (CDC) reported ASD prevalence across all 11 Autism and Developmental Disabilities Monitoring (ADDM) sites, indicating rates per 1,000 children aged eight years ranging from 23.1 in Maryland to 44.9 in California, with an overall prevalence of 27.6 per 1,000 (1 in 36). ASD is 3.8 times more prevalent in boys than in girls (43.0 versus 11.4) [4].

Despite substantial research efforts, the exact etiology remains poorly understood. There are implications of pathological mechanisms combining genetic and non-genetic factors [1]. Recently, increasing evidence has emerged that gut microbial dysbiosis is implicated in the pathogenesis of ASD [5]. The microbiota-gut-brain axis is a tightly regulated, bidirectional communication system that is crucial for maintaining physiological homeostasis [6]. The disruption of the mucosal barrier in the gastrointestinal tract (GIT) can trigger immune responses, resulting in elevated levels of inflammatory cytokines, which compromise intestinal integrity, alter gut microbial composition, and lead to increased gut permeability - sometimes referred to as “leaky gut.” The GIT possesses an intrinsic network of neurons that is affected by this dysbiosis and inflammatory response. Children with ASD experience gastrointestinal (GI) disorders more frequently and suffer from general GI symptoms 4.4 times more often than neurotypical children, with a greater occurrence of diarrhea, constipation, and abdominal pain [6,7].

Despite the increases in research on the gut-brain axis over the past decades, a more comprehensive understanding of the microbiota-gut-brain axis remains essential, particularly regarding the implications of early microbiota dysbiosis and its contribution to the severity of ASD symptoms. Additionally, the impact of maternal microbiome dysbiosis on fetal neurodevelopment is an area that requires further exploration. This review article discusses and examines the current knowledge on the microbiota-gut-brain axis and its role in the development of ASD and symptom severity. The article also explores the pathophysiology of gut dysfunction and outlines potential therapeutic interventions.

## Review

Prevalence of GI symptoms in ASD

ASD patients are four times more likely to develop GI disorders compared to their neurotypical counterparts [8]. The prevalence of GI symptoms ranges from 40% to 90% among ASD patients [9,10]. A meta-analysis of 15 studies revealed that functional GI disorders (FGIDs) are more common in autistic patients than in healthy children [7]. FGIDs are characterized by chronic and recurrent GI symptoms, such as malabsorption and maldigestion, that are not adequately explained by anatomical or biochemical dysfunction. A threefold higher risk of experiencing constipation and diarrhea was reported, along with a twofold higher risk of abdominal pain in autistic children [11]. It is hypothesized that these symptoms stem from microbial overgrowth and increased intestinal permeability, which manifest as constipation, diarrhea, bloating, abdominal pain, reflux, vomiting, flatulence, foul-smelling stools, and food allergies [12,13].

Another study conducted on 1,513 children between 24 to 60 months of age showed that autistic children were almost six to eight times more susceptible to bloating and flatulence, as well as constipation and diarrhea [14]. Leader et al. [15] conducted a systematic review of 30 studies published in the last decade that used caregiver questionnaires to delineate GI symptoms among autistic children. The analysis showed that ASD severity was directly correlated with diarrhea, and parents reported higher food selectivity among autistic children. Constipation and food allergies were also frequently reported [15]. Food selectivity was reported in 62% of the autistic children. Additionally, abnormal stool patterns, such as an increased number of bowel movements per day, increased frequency of unformed bowel movements (more than three per day), and bulky stools, were more common in ASD patients with language regression [16].

Many children with ASD have selective eating habits, preferring processed or carbohydrate-heavy food while avoiding fruits, vegetables, and fiber-rich foods. Autistic children have an altered mechanism of metabolism and absorption of disaccharides from their intestinal epithelium. Therefore, owing to maldigestion in the small intestine, an increased amount of monosaccharides and disaccharides enters the large intestine, where it serves as a substrate for the production of gases, manifesting as bloating and osmotic diarrhea [13]. The results of another study performed on 36 children with autism showed reflux esophagitis in 25 test subjects, chronic gastritis in 15, and chronic duodenitis in 24 patients. Additionally, 21 children showed decreased activity of intestinal digestive enzymes specific to carbohydrates, and 27 showed increased exocrine secretion of pancreatic-biliary fluid after secretin was administered intravenously [17].

Impact of GI symptoms on behavior

There seems to be a bidirectional relationship between the severity of neurobehavioral symptoms and the risk of having GI problems. According to the Autism Treatment Evaluation Checklist (ATEC), an increase in GI symptoms leads to an increase in neurobehavioral symptoms, and vice versa [7]. Children who have both ASD and GI disturbances showcase heightened anxiety, irritability, and social withdrawal compared with those without GI disturbances [18]. The Childhood Autism Risks from Genetics and Environment (CHARGE) study found that GI abnormalities were linked to more consistent autism-related behavior, as assessed by the Aberrant Behavior Checklist [14].

Since there is no objective biomarker for predicting the manifestation of GI symptoms, their onset or worsening is often preceded by the emergence or intensification of certain behavioral issues, which may serve as a child’s way of expressing distress [17]. Increased cortisol in children with ASD has been linked to more severe behavioral and GI symptoms [8]. A link between rigid-compulsive behavior and the presence of constipation has also been delineated [13]. Moreover, GI problems in autistic children have been associated with increased tantrums and aggression. Central nervous system (CNS)-related comorbidities, like seizures, are also frequently observed alongside GI dysfunction in individuals with ASD [8]. Further research has shown that autistic individuals with GI comorbidities experience higher rates of sleep disturbances, abnormal mood, and behaviors such as argumentativeness, oppositional and defiant tendencies, destructiveness, anxiety, sensory sensitivities and responsiveness, rigid-compulsive actions, self-injury, aggression, reduced expressive language, and greater social impairments when compared to autistic individuals without GI issues [19,20].

Alterations in gut microbiota in ASD

The gut environment is predominantly dominated by anaerobic bacteria and other microbials, including viruses, protozoa, archaea, and fungi. *Bacteroidetes* and *Firmicutes* are the two dominating bacterial phylotypes. *Proteobacteria, Actinomyces, Fusobacterium, *and* Verrucomicrobia* comprise a relatively small proportion [21]. Recent clinical studies have focused on the effects of dysbiosis in the gut-brain axis; neurodegenerative diseases such as Alzheimer’s disease and Parkinson’s disease; and conditions such as addiction, anxiety, and depression [22]. Children with ASD have a five times greater risk of having feeding problems. GI dysfunction and food selectivity in ASD have intensified concerns about feeding and the consequent medical sequelae in ASD. Gut microbiota is influenced by age, diet, infection, and stress. An infant inherits the maternal microbiota, which attains maturity by the age of one [23].

A distinct gut flora profile is demonstrated by some studies in ASD compared to neurotypical children [24]. Studies describe a decrease in the abundance of *Bacteroidetes* (9.2% in autistic subjects vs. 19.4% in neurotypical subjects), whereas others show an increase of about 14.3% in children with ASD (95% CI: 12.79, 15.87) compared to 10.9% in the control group. Meanwhile, *Firmicutes* abundance is found to be 13.42% in children with autism (95% CI: 12.50, 14.34) and 10.77% in the control group (95% CI: 9.89, 11.64). However, the *Bacteroidetes* to *Firmicutes* ratio in children with ASD is higher (0.69) than in controls (0.44), along with the abundance of *Alistipes, Bilophila, Veillonella, Collinsella, Corynebacterium*, and *Lactobacillus* [10,25]. In the case of *Clostridium*, more species were found in the feces of children with autism, 0.74% (95% CI: 0.44, 1.05), than in the control group, 0.16% (95% CI: 0.06, 0.26) [10]. A 10-fold increase in *Clostridium* species was found in ASD patients compared to notable healthy control species. *Clostridium bolteae* and *Clostridium* clusters I, II, and XI, which include *Clostridium perfringens* and *Clostridium difficile*, were significantly elevated in ASD patients [26]. In certain studies, the load of *Clostridium* can be correlated with the severity of ASD symptoms [25]. Notably, the *Firmicutes* to *Bacteroidetes* ratio increased with an abundance of *Clostridium*, *Desulfovibrio*, and *Lactobacillus* species [27]. Other studies demonstrated a reduction in beneficial *Bacteroidetes* while showing an increase in *Betaproteobacteria*, often associated with the inflammatory process [28]. *Bifidobacterium*, known as a promoter of healthy status, is proposed to be a psychobiotic owing to its ability to produce neuromodulators and influence the gut-brain relationship. A study reported a decrease in *Bifidobacteria* from 15.302 ± 2.943 among healthy controls to 6.904 ± 2.021 among children with ASD [29].

Factors influencing gut microbiota in ASD

Several factors contribute to the formation of this intestinal microbiota, which starts during the prenatal period. Recent studies have established the presence of microbiome in the placenta, amniotic fluid, meconium, and the blood of the umbilical cord, which indicates that colonization of the GI tract begins during the prenatal period [30]. Human development is known to be rooted in the prenatal stage, and maternal infection during this period can impact the fetus. A substantial correlation between maternal infection during pregnancy and the onset of ASD is supported by epidemiological research. Analysis of infants born to women exposed to the 1964 rubella epidemic provides strong evidence in favor of this theory. This ecological cohort showed an 8%-13% higher frequency of children with autism disorders compared to 0.05% in controls [31,32]. This is consistent with results obtained in maternal immune-activated (MIA) mice. Polyinosinic:polycytidylic acid (poly(I:C)) was administered to pregnant mice to mimic viral infections. The progeny exhibited GI barrier abnormalities and changed microbial composition, resulting in autistic-like behavioral abnormalities. Since poly(I:C) is also a cytokine, it was suggested that elevated cytokine levels in the mother's blood were the cause. The colon of MIA mice had higher than normal amounts of interleukin-6 (IL-6) [33].

The GI microbial colonization of the neonate is impacted by the mode of delivery, preterm birth, and breastfeeding [13]. Infants delivered vaginally are exposed to their mother’s vaginal microbiota, dominated by *Lactobacillus, Prevotella, *and* Snethia* spp., whereas infants born via C-section come into contact with the mother's skin surface bacteria, constituting *Staphylococcus, Corynebacterium, *and* Propionibacterium* spp. [34]. Breastfeeding plays a significant role in formulating gut microbiota during the initial days of life. Breastfed neonates possess the healthiest gut microbiota, mainly composed of *Bifidobacterium* spp., which aid in human milk digestion. A strong correlation has been established between autism and considerably shorter periods of breastfeeding [35]. Preterm birth delays GIT maturity, which leads to longer survival of stomach bacteria that colonize the intestines and alters the gut microbial composition [13]. The normal establishment of microbiota can be harmed by antibiotic treatment during the first three years of life, with long-term consequences. The gut flora of many autistic children may be disrupted by the relatively large dosages of oral antibiotics they receive in their early years. Antibiotics also affect the commensal bacteria that support gut homeostasis [36].

Mechanism

Gut Permeability

The gut-brain axis is a bidirectional physiological connection that enables information exchange between the gut and the brain. A study conducted in autistic mice used metformin to restore, and dextran sulfate to damage, the intestinal barrier. Administration of metformin considerably reduced repetitive and anxiety-related behaviors and promoted social connections among autistic mice [37]. Environmental triggers can lead to neurobehavioral changes by the inappropriate trafficking of the gut-brain axis through inappropriate trafficking caused by an impaired intestinal barrier. In patients with ASD, molecular analysis showed altered blood-brain barrier (BBB) integrity. Increased expression of matrix metalloproteinase-9 (MMP-9) in ASD patients induces BBB disruption, which is a crucial step in the inflammation of the nervous system. About 75% of ASD subjects showed decreased expression of barrier-forming tight junction components like claudin-1, among others, while about 66% showed increased pore-forming claudins [38]. Non-autistic siblings of autistic children exhibit increased gut permeability compared to controls. Furthermore, close relatives of autistic individuals also showed increased intestinal permeability, suggesting that intestinal integrity is not a consequence of ASD but rather a causative factor [20,38].

Altered Metabolites

Bacteria associated with ASD, including *Clostridia, Bacteroidetes, *and* Desulfovibrio*, play an essential role in producing propionic acid (PPA) and other short-chain fatty acids (SCFAs). These readily cross the blood-brain and gut-brain barriers, affecting behavior. When injected in animal models, PPA has been shown to impair behavior and cognition, restrict interest, and induce neuroinflammation. Elevated SCFA and ammonia levels have been found in children with ASD. Some studies have identified molecules from the urine of ASD patients, such as dimethylamine, hippuric acid, and phenylacetylglutamine. Reduced levels of p-hydroxyphenylacetate, a metabolite associated with protective bacteria like *Bifidobacteria *and* Lactobacillus*, have been observed among ASD patients [26,29].

3-(3-hydroxyphenyl)-3-hydroxypropionic acid induces autistic symptoms by decreasing catecholamine levels in the brain [20]. Significantly higher concentrations of 3-(3-hydroxyphenyl)-3-hydroxypropionic acid, 3-hydroxyphenylacetic acid, and 3-hydroxyhippuric acid were found in the analyzed urine samples of autistic children. A significant reduction in the levels of these metabolites was observed - resulting in improved eye contact and reduced constipation - after administration of vancomycin to target colonies of gram-positive bacteria [39]. p-Cresol, a metabolite produced only in the gut, is responsible for neurotransmitter breakdown and is being investigated as a possible biomarker of ASD. Its occurrence correlates with increased gut permeability. p-Cresol is found to be elevated in the urinary and fecal samples of autistic children [13,40].

Significant differences are found in purine, vitamin B6, and phenylalanine-tyrosine-tryptophan biosynthesis and metabolism in autistic children compared to non-autistic children [28,29]. In humans, tryptophan is metabolized by the kynurenine pathway and the 5-hydroxytryptophan pathway. Metabolites of the kynurenine pathway - xanthurenic acid and quinolinic acid - preferentially increased, while serotonin and melatonin, products of the 5-hydroxytryptophan pathway, decreased. The modified tryptophan metabolism, influenced by gut microbiota, led to elevated levels of indolyl-3-acetic acid and indolyl-lactate. Disrupting the gut microbial colonies impacts vitamin levels in the body. Kynureninase, an enzyme in the kynurenine pathway, utilizes vitamin B6 as a cofactor. Therefore, vitamin B6 deficiency results in low concentrations of kynurenic acid and elevated levels of xanthurenic and quinolinic acids [13,40,41]. *Escherichia coli*, *Proteus vulgaris*, *Paracolobactrum coliforme*, *Achromobacter liquefaciens*, *Bacteroides*, and *Clostridium* spp. are the bacteria that produce indoles. Higher levels of indole and 3-methylindole were detected in the feces of autistic children. Elevated levels of indolyl-3-acetic acid and indolyl-lactate were found in the urine. Further research is needed to assess the true potential of these metabolites as biomarkers in ASD [40,42].

Neural Pathway

Neurotransmitters, including gamma-aminobutyric acid (GABA), histamine, norepinephrine, acetylcholine, serotonin, dopamine, and melatonin, produced by microbiota, directly modulate the vagus nerve. An analysis of serotonin levels as a continuous variable suggested a strong correlation with GI symptoms. Bowel movements, stool retention, and abdominal pain were linked to greater serotonin levels [43]. The autonomic nervous system (ANS), via neurons, smooth muscle cells, epithelial cells, and immune cells, directly modulates gut motility, permeability, mucus production, bile secretion, and intestinal osmolarity [44]. Table 1 summarizes the microbiota alterations and their impact on neurotransmitters [45].

**Table 1:** Effect of gut dysbiosis on neurotransmitters levels Table credit: [45] GABA, gamma-aminobutyric acid

Microorganism	Neurotransmitter	Effect on the levels
Lactobacillus	GABA, acetylcholine, norepinephrine, and dopamine	Decrease
Bifidobacterium	GABA	Decrease
Escherichia	Norepinephrine and serotonin	Decrease
*Saccharomyces* spp	Norepinephrine	Increase
Candida	Serotonin	Increase
*Streptococcus* and *Enterococcus*	Serotonin	Decrease
Clostridium	Tryptophan	Increase

Immune Dysfunction

In ASD patients, the associated immune dysfunction of cytokines such as IL-6 and IL-1β, and migration inhibitory factor (MIF), is linked to impaired social communication. Rising circulating lipopolysaccharides (LPS), generated from bacteria, are one effect of enhanced intestinal permeability. This translates to the fact that higher levels of systemic pro-inflammatory cytokines indicate an inflammatory and immunological response [26]. Since many immunological abnormalities have been documented in those with ASD, it has long been known that immune dysfunction may play a role in the condition. Children with autism, particularly those with the regressive form of the illness, have been found to have elevated cytokine levels. In ASD, elevated plasma levels of pro-inflammatory cytokines, including IL-1β, IL-6, IL-8, and IL-12p40, together with MIF and platelet-derived growth factor (PDGF), have been recognized. Elevated concentrations of these cytokines in the bloodstream have been associated with communication difficulties and decreased social interactions [46]. Another crucial mechanism that has been found to heighten stereotypy, hyperactivity, and communication impairments is modification in peripheral immunological markers, including transforming growth factor-beta (TGF-β), P-selectin, and MIF. One revolutionary discovery in the field of ASD research is the neuroinflammatory process observed in postmortem brain samples of individuals with ASD. A comparative study of postmortem and cerebrospinal fluid (CSF) samples from individuals with ASD and healthy controls showed a neuroinflammatory response, characterized by high levels of pro-inflammatory cytokine profiles and excessive activation of microglia [47]. It has been shown that microglia, which are phagocytic cells of the CNS, are essential for immunological surveillance, synaptogenesis, and the developmental apoptosis required for normal CNS maturation [48].

Epigenetics

**Figure 1:**
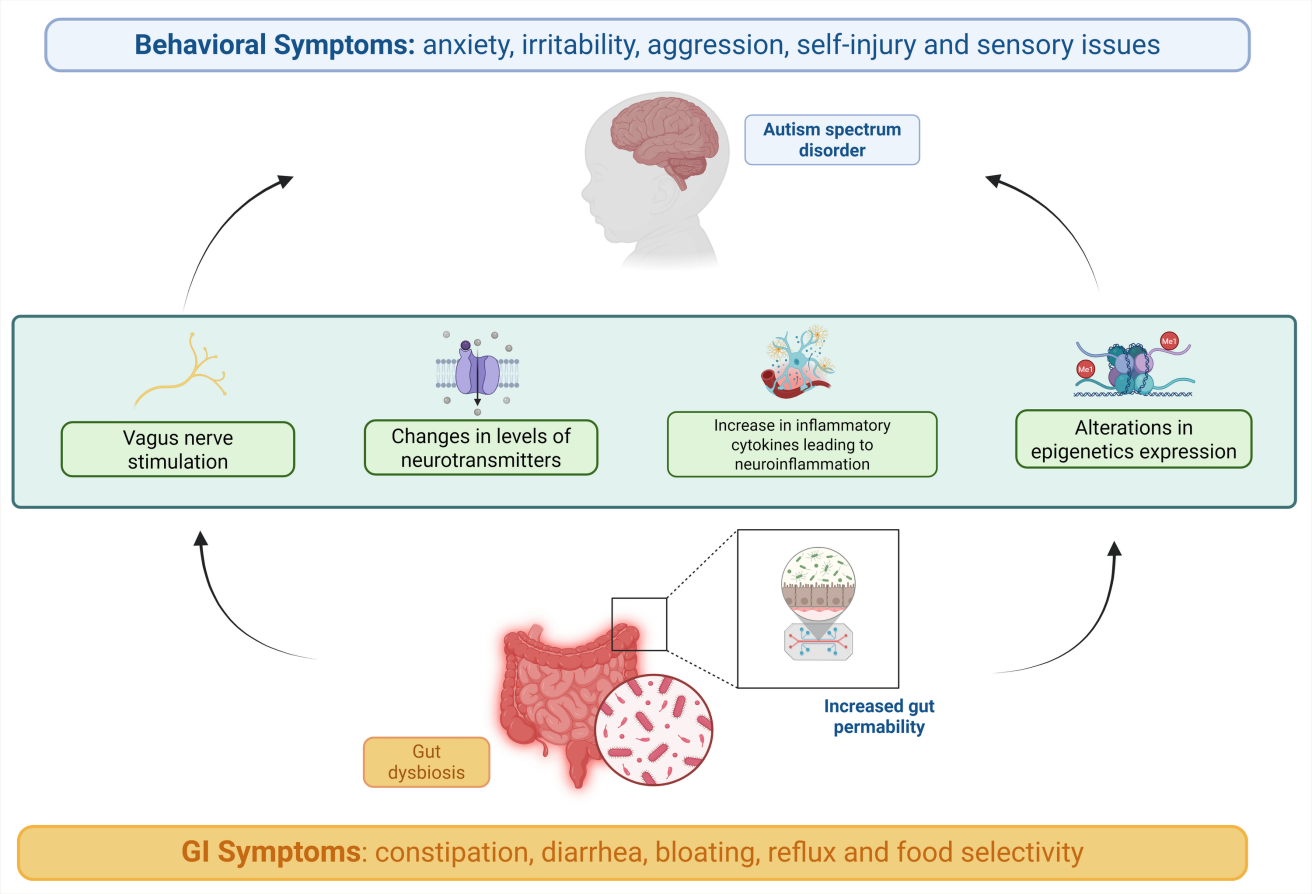
Overview of the pathogenesis of gut dysbiosis and autism spectrum disorder Image credit: Humza Siddiqui and Laura Marrelli; image created using BioRender.com

Environmental factors, including gut dysbiosis, have been shown to cause epigenetic changes leading to autism. A variety of mechanisms, including DNA methylation, histone modifications, and miRNA, have been postulated to play a role in altering epigenetic expression [49]. Research has shown that a reelin gene (RELN) mutation (rs362691) might be a cofactor in the increased risk of ASD. The RELN gene protein plays a key role in neural migration to the neocortex [50]. Other genes affected by methylation and implicated in the initiation of ASD include the engrailed-2 gene (En2), SHANK3 (SH3 and multiple ankyrin repeat domain protein 3), retinoic acid-related orphan receptor alpha (RORA), B-cell lymphoma 2 (BCL2), and ubiquitin protein ligase E3A [49]. Similarly, enhanced expression of miR-557 and miR-486 has been observed among autistic individuals, impacting cognitive function [51]. An overview of the pathogenesis is provided in Figure 1.

Therapeutics

Prebiotics and Probiotics

A prebiotic is defined as a substrate that is selectively utilized by host microorganisms, conferring a health benefit. The therapeutic use of prebiotics is considered advantageous due to their low side effect profile and high tolerability [52]. Though limited studies are available, there are promising effects of prebiotics on the improvement of symptomatology in ASD. Studies using prebiotics as a standalone treatment - such as bovine colostrum products and partially hydrolyzed guar gum - have shown positive effects in reducing GI symptoms in individuals with ASD [52,53]. In contrast, Grimaldi et al. [54] reported no significant improvement in GI symptoms or social behavior in ASD following a six-week intervention with Bimuno® galacto-oligosaccharide (B-GOS). However, a significant synergistic impact was reported when the intervention was combined with an exclusion diet, including a reduction in social withdrawal and an increase in *Bifidobacterium longum*, a species shown to reduce stress and improve memory [54]. Inoue et al. conducted a study analyzing the efficacy of prebiotic water-soluble fiber in ASD patients. The prebiotic supplementation substantially altered gut dysbiosis, improved constipation, and reduced behavioral irritability among autistic children [53]. Therefore, the true effect of prebiotics on ASD remains inconclusive, underscoring the need for more randomized controlled trials with larger sample sizes and assessments by trained clinicians to further evaluate their therapeutic efficacy. 

Probiotics, live microorganisms, play a crucial role in maintaining a healthy gut. These beneficial bacteria, such as *Lactobacillus* and *Bifidobacterium*, are commonly found in fermented foods, powders, and capsules. Probiotics have been used to manage various conditions, including antibiotic-associated diarrhea and irritable bowel disease. There is growing interest in the therapeutic potential of probiotics for ASD, as evidence suggests a complex relationship between the gut microbiome, the brain, and the potential impact of probiotics on this relationship. Anecdotal reports suggest positive benefits, such as alleviation of GI symptoms and improvement in behavior in children with ASD [55]. Reinforcing this notion, Abdellatif et al. reported that, although the efficacy of probiotic supplementation for ASD is promising, the path forward requires more standardized clinical trials to definitively assess their effectiveness and optimal usage in the ASD population. However, Abdellatif et al. also emphasized the need for more clinically controlled trials to adequately assess their true efficacy [56].

Song et al. conducted a meta-analysis that focused specifically on controlled clinical trials to investigate the efficacy of probiotics and prebiotics in reducing the overall severity of ASD symptoms and GI problems in children. It revealed that probiotics and prebiotics do not significantly improve the severity of ASD, GI symptoms, or related mental health issues in individuals with ASD [57]. Sanctuary et al. conducted a double-blinded trial to assess the therapeutic efficacy of prebiotic-probiotic supplementation. *Bifidobacterium infantis* was used as the probiotic, and bovine colostrum product was used as the prebiotic. The supplementation improved both GI and aberrant behavioral symptoms, which were potentially attributed to a decrease in IL-13 and tumor necrosis factor-alpha (TNF-α) among patients [58]. Wang et al. analyzed the therapeutic impact of probiotic and fructo-oligosaccharide (FOS) by measuring the gut microbiota, fecal SCFA, and plasma neurotransmitters. Probiotic and FOS supplementation increased the bacterial colonies of favorable bacteria and reduced pathogenic bacteria such as *Clostridium*. A substantial reduction of acetic acid and PPA and elevated serotonin levels were observed in the intervention group. Significant improvement in the severity of autistic and GI symptoms was found as a result [59].

Fecal Microbiota Transplant (FMT)

FMT has been proven quite beneficial in the long-term improvement of behavioral and GI symptoms of ASD. FMT involves the transfer of diverse bacterial strains from a screened, healthy donor to the recipient, aiming to restore gut microbiota balance. The standardized human gut microbiota is administered either via an enema or a palatable, orally administered drink [60]. In 2019, the Food and Drug Administration (FDA) acknowledged microbial transplant therapy and designated it as a “fast-track” treatment for ASD following successful clinical trials demonstrating the effectiveness of long-term microbial transplant therapy in children with autism [5].

Its use has been shown to be significantly efficient in rebalancing gut microbiomes for recurrent *C. difficile* infection. In a groundbreaking study by Kang et al. [61], 18 participants with ASD and 20 neurotypical participants were followed along a 10-week trial encompassing two weeks of vancomycin, followed by an eight-week trial of FMT. Remarkably, 16 of the 18 participants with ASD showed a remarkable reduction of 82% in GI symptoms, along with vast improvements in behavioral symptoms of ASD. This study further explored the composition of gut microbiomes, revealing that after transplantation, there was a much more diverse gut microbiome composed of *Bifidobacterium, Prevotella, *and* Desulfovibrio*. *Bifidobacterium* increased fourfold compared to its abundance in neurotypical children, which further demonstrated the relation between gut microbial diversity and ASD. These improvements were sustained during the eight-week and two-year follow-up periods. Some adverse effects reported were mild to moderate hyperactivity and manifestations of aggressive behavior at the initiation of vancomycin treatment [35,61]. The FMT improved the epithelial cell lining and permeability of the gut, which led to negligible absorption of fecal metabolites, resulting in relatively normal levels of metabolites in the plasma [61]. Similarly, in a study performed by Li et al., GI and ASD symptoms improved following treatment for four weeks without pre-treatment with vancomycin, and were sustained in an eight-week follow-up [62]. In comparison, Huang et al. reported improvements in GI, ASD, and other psychological symptoms in an adult with Asperger's after a three-week FMT treatment. Although the improvement was sustained for one month, it was not maintained at the three-month follow-up, which was possibly attributed to a personal stressor. This suggests that the method and duration of FMT can play a role in the effectiveness and longevity of results [63].

The dose for each course of FMT is based on the weight of both the donor stool sample and the body weight of the patient receiving the FMT. The usually prescribed ratio is 1 gram of donor stool per 1 kilogram of the recipient’s body weight [64]. It was reported that patients who received intensive microbiota transplantation (IMT) experienced a significant decrease in abdominal cramps, which led to an improvement in their sleeping patterns. Additionally, the parents noticed that the odor of the stool also showed significant improvement. The children showed enhanced empathetic emotional behavior at the end of the study. They also exhibited the ability to listen to and obey the commands of their parents and perform the necessary tasks. There was an improvement in speech as well [65].

Antibiotics

Antibiotics deplete specific groups of bacteria and are commonly used in the treatment of infections in the body. By their mode of action, they can alter gut microbiome diversity to theoretically positively impact ASD. A further advantage is attributed to their ability to titrate doses and form cocktails to achieve the targeted balance of microbiota or desired gut diversity [66]. Albeit this knowledge, no recent studies or trials have illustrated this; merely anecdotal evidence and observations prevail. However, recent available cases illustrate that improvements in GI and ASD symptomology were mere incidental observations reported during antibiotic therapy for a subsequent infection. Unfortunately, these improvements did not sustain long-term [67]. The potential disadvantage of the emergence of antibiotic-resistant bacteria due to long-term use, along with harmful side effects, limits studies on this therapeutic avenue [68].

Zinc Supplementation

Zinc deficiency is a common finding in individuals with ASD. It is also observed in individuals with attention deficit hyperactivity disorder (ADHD) and depression. Zinc deficiency can cause problems related to GI function, resulting in other micronutrient deficiencies. ASD involves a complex interplay of genetic and environmental factors, with zinc playing a crucial role. Sterile alpha motif (SAM) is one key component in the SHANK3 protein, providing scaffolding essential for the formation and function of synapses. SHANK3, with its zinc-binding site, provides post-synaptic support and is responsible for synaptic transmission [69]. Studies have revealed that zinc plays a critical role in regulating the activation of SHANK3, a key player in synaptic transmission. SHANK3, with its zinc-sensitive signaling system, orchestrates the flow of information across synapses. Disruptions in this zinc-sensitive signaling system - often observed in individuals with ASD carrying SHANK3 mutations - can lead to impaired synaptic function and plasticity, potentially contributing to the behavioral characteristics associated with ASD [70]. Intriguingly, increasing dietary zinc in mouse models of ASD has shown promising results, reversing ASD-related behaviors in young mice and their offspring. This suggests that dietary zinc supplementation may hold potential as a therapeutic strategy for ASD. However, the precise mechanisms by which dietary zinc exerts its beneficial effects remain a subject of ongoing research. One intriguing possibility is that dietary zinc may influence the gut microbiome and gut permeability, potentially contributing to its positive effects on ASD-related behaviors. Further research is needed to unravel the complex interplay between zinc, the gut, and ASD, paving the way for novel therapeutic interventions [71].

Dietary Interventions

Children with ASD consume fewer vegetables and tend to prefer energy-dense foods, resulting in insufficient fiber intake. It is crucial to examine dietary patterns, as they may contribute to imbalances in gut microbiota composition and GI issues. The most extensively researched nutritional strategies in the literature include the gluten-free/casein-free diet (GF/CFD), ketogenic diet (KD), specific carbohydrate diet (SCD), and Mediterranean diet (MD) [72].

The GF/CFD is a dietary intervention frequently considered for individuals with ASD. While research on healthy individuals has demonstrated that GF diets can negatively impact gut microbiota by reducing beneficial bacteria populations, increasing opportunistic pathogens, and potentially inducing immune suppression, the application of this diet in ASD remains a subject of debate [72]. The GF/CFD may be recommended when an allergy or intolerance to gluten or casein is diagnosed. However, the evidence regarding its efficacy in ASD is conflicting. Some studies have reported positive outcomes, suggesting that the GF/CFD can reduce urine peptides, improve behavioral symptoms, and alleviate GI symptoms. Conversely, other studies have highlighted potential drawbacks, such as decreased fiber intake, which could exacerbate existing GI problems [73,74]. The current body of evidence supporting or refuting the use of GF/CFD in ASD is limited and inadequate. This inadequacy stems from the insufficient quantity and quality of research, as well as the presence of multiple methodological limitations in existing studies. Further rigorous and well-designed research is necessary to definitively assess the efficacy and safety of the GF/CFD in individuals with ASD [72].

The KD is a high-fat, low-carbohydrate regimen that has proven effective in treating epileptic patients who don't respond to conventional anticonvulsant medications. Its potential benefits extend beyond epilepsy, with research exploring its application in various conditions, including ASD [72]. In individuals with ASD, the KD has shown promise, particularly in mild to moderate cases. Reports suggest improvements in seizure symptoms and behavioral deficits [75]. Animal studies provide biological insights into the KD's effects. In the BTBR T+Itpr3tf/J mouse model of ASD (BTBR), the KD demonstrated improvements in behavioral deficits associated with ASD, such as sociability, repetitive behaviors, and social communication [76,77]. Furthermore, KD treatment in BTBR mice mitigated deficits related to myelin formation, white matter development, and connectivity, influencing neurotransmitter signaling pathways involving glutamate, serotonin, neuronal nitric oxide synthase, and dopamine. Interestingly, BTBR mice exhibited a distinct gut microbiota profile compared to control mice [77,78]. However, the KD is not without its disadvantages. It is linked to increased inflammatory risk, mitochondrial dysfunction, and potential side effects, like constipation and reflux, which could exacerbate GI comorbidities in ASD. A systematic review of the KD in ASD concluded that the limited number of reports on its effectiveness is insufficient to support its widespread use as a treatment for the disorder [75].

**Figure 2:**
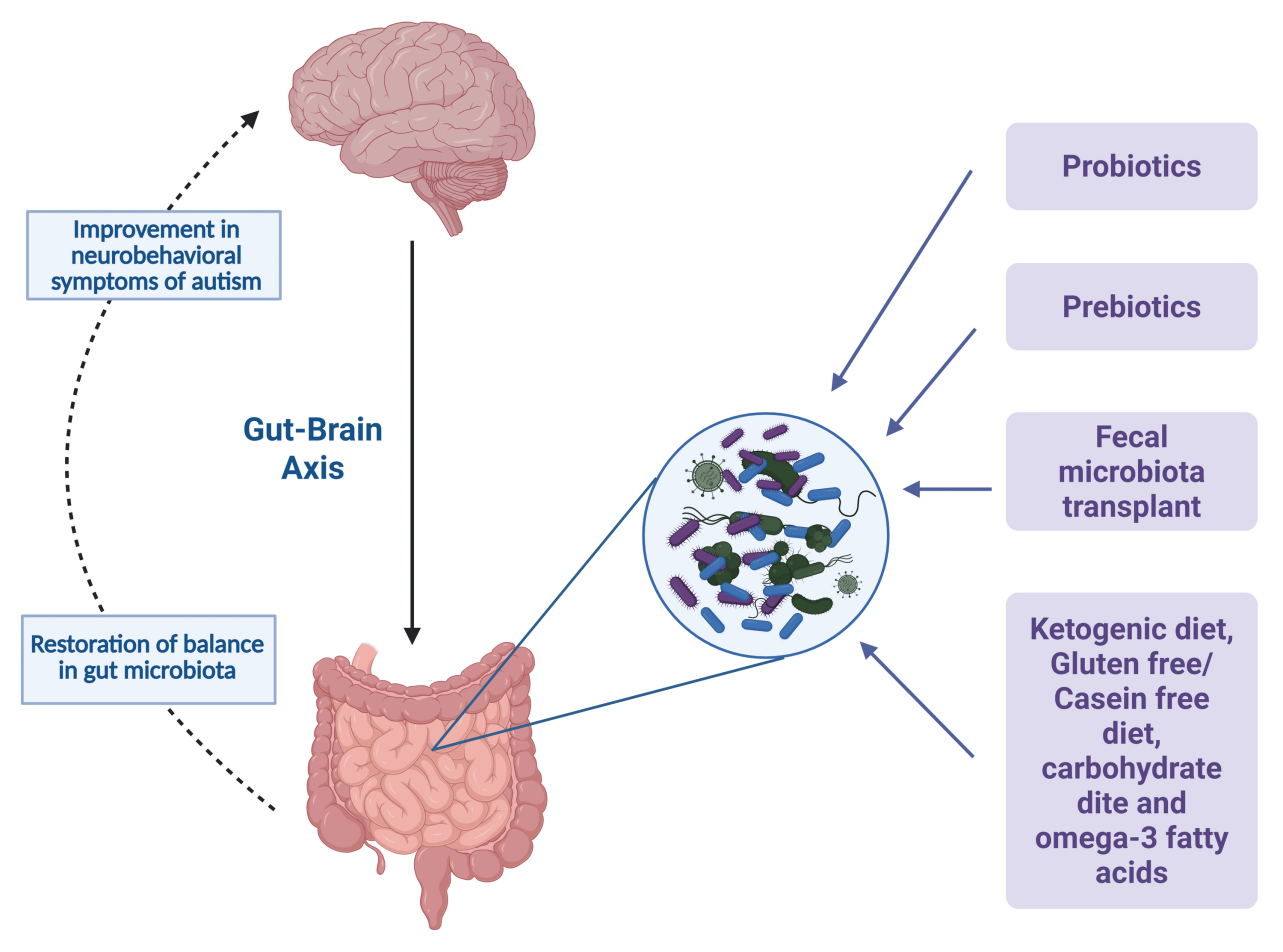
Overview of therapeutic strategies Image credit: Humza Siddiqui and Laura Marrelli; image created using BioRender.com

SCFAs are produced from the microbial fermentation of plant-based fiber. Their role in the regulation of the gut microbiota needs to be established. Because of SCFA, the oxygen concentration decreases due to an increase in oxidative respiration, which further leads to stabilization of the hypoxia-inducible factor 1α (HIF-1α). This blocks the movement of gut microbiota and shields the microorganisms from the host's immune defense. Research on dietary fat intake indicates that consuming a high-fat diet lowers the levels of SCFAs, such as butyrate, and decreases the population of *Bifidobacteria*. However, while a high-fat diet may help reduce intestinal inflammation, it also leads to increased levels of inflammatory markers in the blood and higher circulation of LPS [5]. The supplementation of 1,000 mg of omega-3 showed improved stereotyped behavior compared to the controls [79]. The at-risk preterm group displayed an improvement in interpersonal relationship skills compared to the controls after administration of omega-3-6-9 fatty acids [80]. Therapeutic strategies have been summarized in Figure 2.

## Conclusions

The evident differences between the gut microbiomes of neurotypical people and ASD patients highlight the profound impact that microbial colonies have on immunological response, behavior, and cognition. According to current research, therapies that primarily address gut dysbiosis - including FMT and dietary supplementation - have shown promise in modifying ASD symptoms by rebalancing neurotransmitter production, lowering inflammation, and restoring microbial balance. The intricacy of the gut-brain-microbiota axis necessitates careful research, and extensive clinical trials are required to confirm the effectiveness of these treatments. The increasingly widespread understanding of the gut's role in ASD signals a paradigm shift in therapeutic approaches, emphasizing precision medicine and microbiome-based interventions. However, the search for a long-term cure is still underway and requires extensive research.
